# Ankylosing spondylitis complicated by cervical spine fracture and cervicothoracic epidural hematoma: A case report

**DOI:** 10.1097/MD.0000000000046203

**Published:** 2025-12-26

**Authors:** Yongbo Li, Huilin Feng, Deyuan Chen, Jianqiang Yan, Fuli Huang

**Affiliations:** a Graduate School, Guangzhou University of Chinese Medicine, Guangzhou, China; b Zhongshan Hospital of Traditional Chinese Medicine Affiliated to Guangzhou University of Chinese Medicine, Zhongshan, China.

**Keywords:** ankylosing spondylitis, cervical spine fracture, epidural hematoma, hematoma evacuation, spinal cord decompression

## Abstract

**Rationale:**

Patients with ankylosing spondylitis (AS) are uniquely susceptible to severe cervical spine fractures and spinal epidural hematoma (SEH) following minor trauma, leading to rapid neurological decline. The distinct biomechanical and vascular pathophysiology in AS necessitates tailored surgical management strategies.

**Patient concerns:**

A 68-year-old man with a known history of AS presented with acute neck pain and progressive weakness in both lower limbs.

**Diagnoses:**

Computed tomography and magnetic resonance imaging revealed a fracture at the C5–7 level, accompanied by an extensive cervical epidural hematoma causing significant spinal cord compression. The diagnoses were cervical spine fracture with incomplete spinal cord injury and traumatic SEH.

**Interventions:**

Due to rapid neurological deterioration culminating in grade 0 muscle strength in the lower limbs, urgent surgical intervention was performed. A combined anterior–posterior approach was utilized: anterior C7 subtotal corpectomy with fusion for stabilization, followed by posterior laminectomy for decompression. A novel technique involving gentle evacuation of the hematoma using a specialized dural catheter was employed to restore spinal cord pulsation.

**Outcomes:**

Postoperatively, the patient demonstrated significant neurological recovery, achieving grade 4/5 muscle strength in the lower limbs and the ability to walk with a walker. Two-year follow-up confirmed stable instrumentation, normal limb strength, and only transient residual plantar numbness. No major complications such as infection, cerebrospinal fluid leak, or hardware failure occurred.

**Lessons:**

Early diagnosis and urgent surgical decompression are critical for neurological recovery in AS patients with cervical fractures and SEH. The combined anterior–posterior approach provides effective decompression and robust stabilization. The technique of limited laminectomy with catheter-assisted hematoma evacuation represents an effective minimally invasive strategy for decompression while preserving posterior spinal integrity.

## 1. Introduction

Ankylosing spondylitis (AS) is a chronic inflammatory autoimmune disorder primarily affecting the axial skeleton, characterized by progressive ossification of spinal ligaments, facet joints, and intervertebral discs, leading to a rigid “bamboo spine” phenotype. This structural transformation, coupled with systemic osteoporosis, significantly compromises spinal biomechanical integrity, rendering the spine highly susceptible to fractures even after minor trauma.^[1]^ Advanced AS is associated with reduced bone mineral density at key sites such as the lumbar spine and hip, further elevating fracture risk.^[2,3]^ The pathophysiological cascade in AS involves chronic inflammation driven by cytokines such as TNF-α, which not only promotes ectopic bone formation but also contributes to vascular fragility and endothelial dysfunction.^[4]^ These changes increase the likelihood of epidural vascular rupture following spinal fracture, leading to spinal epidural hematoma (SEH). SEH is a rare but serious complication, with an estimated incidence of 0.1 per 100,000 in the general population, but occurring in up to 21.6% of AS patients with spinal fractures.^[5]^ Cervical fractures in AS often involve multiple segments and traverse all 3 spinal columns, resulting in highly unstable “hinge-type” injuries. The cervicothoracic junction is particularly vulnerable due to its transitional biomechanics and stress concentration. Even minimal displacement can rupture the inelastic para-spinal venous plexus or arterial branches, leading to rapid SEH formation and acute neurological deterioration.^[6]^ Moreover, the ossified epidural space in AS offers little compliance, allowing even small hematomas to cause significant spinal cord compression. Early diagnosis is challenging due to preexisting spinal deformity and chronic pain, which may mask acute symptoms. Imaging plays a critical role, with CT being superior for detecting fracture morphology and MRI essential for evaluating neural compression, ligamentous injury, and SEH.^[7]^ Recent studies emphasize the importance of whole-spine CT with 3D reconstruction for fracture detection and MRI for neurological assessment, particularly in lower cervical injuries where SEH is more common.^[8]^

Given the complexity of AS-related spinal injuries, a multidisciplinary approach involving spine surgery, rheumatology, radiology, and rehabilitation is essential for optimal outcomes. In January 2023, our institution managed a case of AS complicated by cervical fracture and cervicothoracic SEH. The patient underwent short-segment laminectomy decompression with customized dural soft catheterization and syringe-assisted irrigation to evacuate hematomas through the spinal canal. Favorable postoperative recovery was observed during follow-up. This experience informs the development of minimally invasive laminotomy techniques for SEH clot evacuation, as systematically detailed in this report.

## 2. Case presentation

A 68-year-old male Macau resident presented to our institution with neck pain and bilateral lower extremity weakness persisting for 4 hours post-fall. The patient had been diagnosed with ankylosing spondylitis a decade prior but had not received standardized disease-modifying therapy. Comprehensive diagnostic workup confirmed: cervical vertebral fractures (C5–C7) with incomplete spinal cord injury; cervicothoracic spinal epidural hematoma; advanced ankylosing spondylitis; and stage II hypertension.

**Figure 1.**
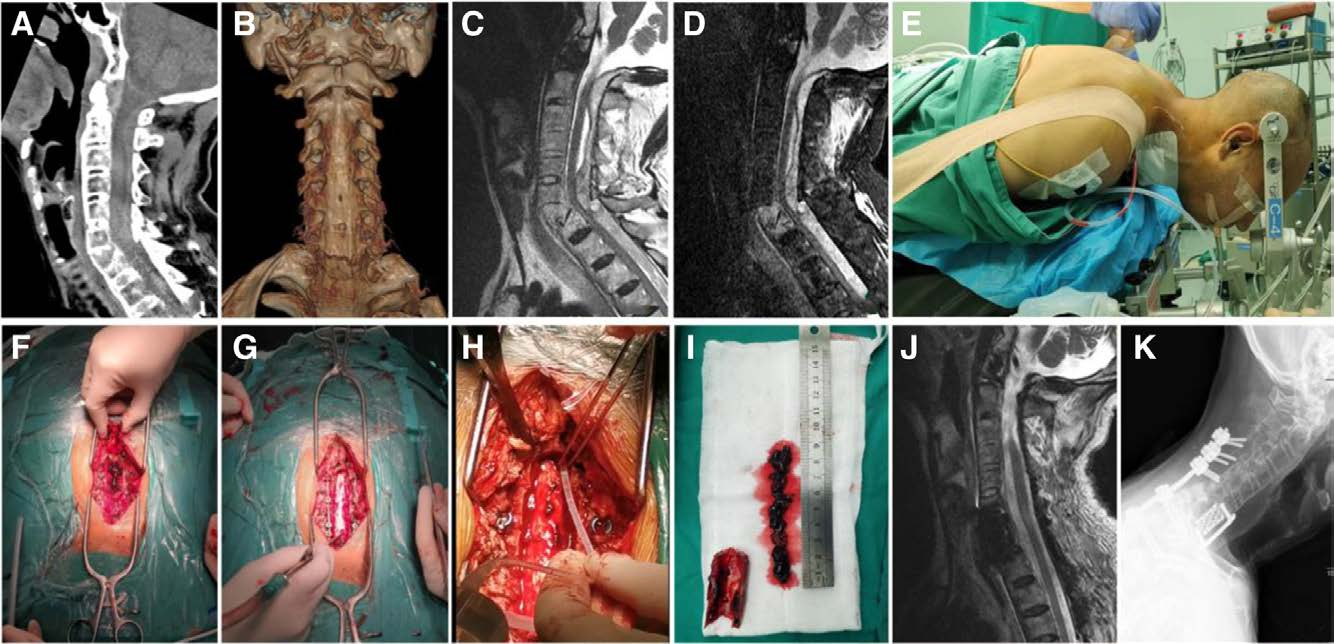
(A–B) Preoperative cervical CT scans: (A) Sagittal reconstruction demonstrates a fracture through the C7 vertebral body with associated hyperextension deformity and loss of normal cervical lordosis. Note the extensive syndesmophyte formation and ankylosis of facet joints, characteristic of advanced ankylosing spondylitis. (B) Axial image reveals comminuted fractures of the anterior elements at C7; C–D, Preoperative MRI: (C) Sagittal T2-weighted image shows a extensive posterior epidural hematoma extending from the mid-cervical to the upper thoracic spine, causing severe compression and displacement of the spinal cord. (D) Axial T2-weighted image at the C6/7 level confirms the significant cord compression and signal changes within the cord suggestive of contusion and edema; (E) Intraoperative positioning: The head was stabilized using a Mayfield head frame to ensure precise alignment and stability during the posterior procedure; (F) Intraoperative exposure: After laminectomy, dark red clots are seen covering the dorsal surface of the dura mater; (G) Decompressed spinal cord: Following hematoma evacuation, the spinal cord shows restored pulsation and adequate decompression; (H) Irrigation technique: A customized dural soft catheter connected to a syringe is being used to gently irrigate and aspirate hematoma from the spinal canal; (I) Evacuated hematoma: The aspirated clot, measuring approximately 9 cm in length; (J) Postoperative MRI: Sagittal T2-weighted image obtained prior to discharge confirms complete evacuation of the epidural hematoma and adequate decompression of the spinal canal; (k) 6-month follow-up X-ray: Lateral cervical spine radiograph demonstrates maintained alignment and stable positioning of the anterior titanium mesh cage and posterior instrumentation without signs of loosening or migration.

Physical examination revealed loss of cervical lordosis with restricted range of motion and tenderness over the cervicoscapular region. Sensory impairment was noted as hypesthesia below the T2/T3 dermatomal level (corresponding to the sternal notch), accompanied by perianal anesthesia (in the S3–S5 dermatome distribution). Motor assessment demonstrated grade 4 strength in right wrist extensors (radial/ulnar), biceps, brachioradialis, triceps, and wrist flexors, while other right upper extremity muscles maintained grade 5 strength. Left upper extremity exhibited full (grade 5) strength throughout. Bilateral lower extremities showed severely diminished motor function (grade 1+). Normal muscle tone was preserved in all limbs. Neurological evaluation identified negative Hoffmann signs bilaterally with absent cremasteric and anal reflexes, accompanied by neurogenic bladder/bowel dysfunction. The patient’s medical history included essential hypertension and seropositive ankylosing spondylitis, with no documented diabetes mellitus, cardiovascular diseases, neurospinal pathologies, significant trauma, or prior surgical interventions. Diagnostic workup revealed HLA-B27 seropositivity. Inflammatory markers showed elevated C-reactive protein (11.99 mg/L) and markedly increased D-dimer levels (>20.00 μg/mL). Cervical CT imaging identified a C7 vertebral body fracture with multi-level posterior element fractures from C5-C7, accompanied by hyperextension deformity and C6 segmental instability. Characteristic AS-related changes including syndesmophyte formation and facet joint ankylosis were evident(Fig. 1A,B). Magnetic resonance imaging disclosed a longitudinal posterior epidural hematoma extending through cervical to upper thoracic regions, causing significant spinal cord compression with signal changes indicative of contusion and edema at C6–C7 levels (Fig. 1C,D). Ancillary evaluations including electrocardiography, echocardiography, cardiac biomarkers, chest radiography, and cranial CT demonstrated no acute cardiopulmonary or intracranial abnormalities.

Preoperative preparation involved cervical collar immobilization and routine symptomatic management including anti-inflammatory analgesic medications, dehydration therapy, hemostasis, muscle relaxation, and gastric protection, aiming to reduce spinal cord compression and suppress inflammatory responses. The patient remained afebrile and normoxic throughout preoperative and postoperative periods. Rapid motor decline occurred post-admission, with preoperative bilateral lower limb strength reaching grade 0, necessitating emergent surgical decompression.

**Figure 2.**
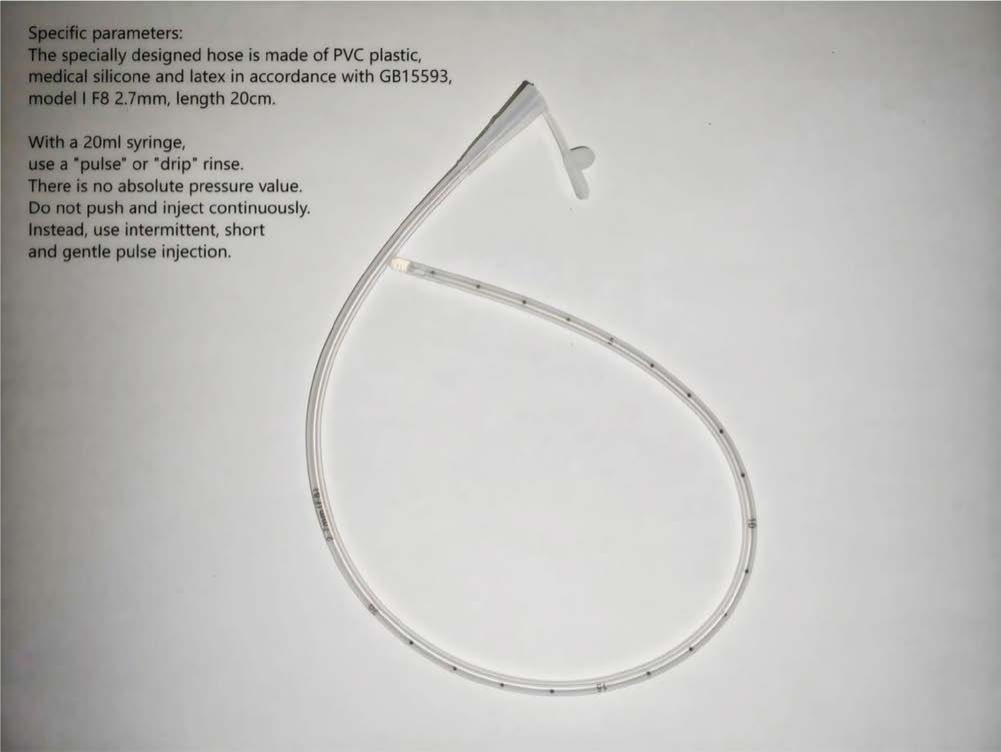
Specific parameters: The specially designed hose is made of PVC plastic, medical silicone and latex in accordance with GB15593, model I F8 2.7 mm, length 20 cm. With a 20 mL syringe, use a “pulse” or “drip” rinse. There is no absolute pressure value. Do not push and inject continuously. Instead, use intermittent, short and gentle pulse injection.

Under general anesthesia with endotracheal intubation, an initial anterior cervical approach was performed for C7 subtotal corpectomy and fusion. Surgical exposure revealed a comminuted C7 vertebral body with severe intravertebral bone loss. Rongeurs and a high-speed burr were used to perform the subtotal corpectomy. A titanium mesh cage filled with autograft was implanted for support and fixation, followed by placement of an anterior cervical plate-screw system. After completing the anterior procedure, the patient was repositioned prone for posterior cervical laminectomy with instrumented fusion and evacuation of the intraspinal hematoma (Fig. 1E). Surgical exposure was achieved, and an ultrasonic bone scalpel was used to divide the bilateral laminae. Upon elevation of the laminae, dark red clots were observed covering the spinal cord surface (Fig. 1F). Significant dural compression with absent CSF pulsation was noted (Fig. 1G). A customized dural soft catheter (composed of medical-grade polyvinyl chloride complying with GB15593, silicone, and latex; Type I, F8 [2.7 mm in diameter], 20 cm in length; primarily intended for postoperative drainage and compatible with drainage bags or bottles) (Fig. 2) connected to a syringe irrigation system was gently advanced into the spinal canal to evacuate the clots (Fig. 1h). Under controlled low-pressure saline irrigation, substantial dark red clots were aspirated (Fig. 1i), followed by restoration of dural pulsation. Lateral mass screws were implanted at the midpoints of C4-6. Bilateral pedicle screws were placed at T1-2. Rods were connected to achieve fixation. Hemostasis was confirmed before wound closure. The total operative time was 300 minutes, with an estimated blood loss of 400 mL.

Postoperative rehabilitation was initiated on the first day following surgery. Passive joint mobilization of all 4 limbs was commenced within 24 hours, administered twice daily for 20 minutes per session, with intensity carefully regulated to maintain full range of motion without provoking pain. By the second week, active-assisted exercises were introduced to strengthen proximal musculature of the upper and lower limbs, alongside gait training with a walker as lower extremity motor strength improved to grade 3/5. Pre-discharge imaging confirmed satisfactory positioning of the pedicle screws and titanium mesh cage, along with complete evacuation of the spinal epidural hematoma (Fig. 1j). Neurological assessment at discharge revealed preserved upper extremity strength (grade 5) and improved lower limb motor function (grade 4–5), enabling assisted ambulation. Sensory impairments included bilateral hypesthesia below the L1 dermatome and persistent saddle anesthesia, while plantar dysesthesia was noted alongside regained voluntary sphincter control. At the 6-month follow-up, radiographic evaluation demonstrated stable instrumentation without evidence of hardware migration (Fig. 1k), accompanied by full motor recovery despite residual plantar paresthesia. The final 24-month assessment confirmed sustained neurological recovery, with complete resolution of plantar sensory deficits and maintained functional independence.

## 3. Discussion

Cervical fractures complicated by SEH in AS patients represent a highly challenging clinical emergency. AS patients’ spinal fusion, osteoporosis, and biomechanical fragility predispose them to multi-segment fractures from minor trauma, often accompanied by paraspinal vascular rupture leading to SEH formation.^[9]^ In the present case, the patient sustained a comminuted C7 vertebral fracture combined with extensive posterior cervicothoracic epidural hematoma, accompanied by rapid neurological deterioration requiring urgent surgical intervention. AS patients exhibit “bamboo-like” spinal changes where bony fusion causes stress concentration, while osteoporosis further compromises bone strength. Studies indicate that spinal fractures in AS patients frequently involve 3-column structures, with fracture lines traversing vertebral bodies and posterior elements, creating “hinge-type” unstable injuries.^[3]^ Such fractures are prone to cause epidural hematomas due to minor displacement stretching paraspinal venous plexuses or arterial branches. CT findings in this case revealed C5-7 posterior element fractures and posterior column soft tissue injury, confirming 3-column damage mechanism and indicating the need for long-segment fixation to restore stability. Beyond mechanical vascular tearing, chronic inflammation in AS patients may exacerbate vascular fragility. Studies show inflammatory factors like TNF-α can compromise vascular endothelial integrity, elevating spontaneous hemorrhage risk.^[4]^ Additionally, the spinal epidural space in AS patients becomes narrowed due to ligament ossification, making hematomas more likely to compress the spinal cord. MRI in this case revealed extensive continuous hematoma compression of the dural sac in cervicothoracic regions, closely related to spinal stenosis, highlighting the importance of early imaging evaluation for defining hematoma extent. For AS patients with cervical fractures and SEH, combined anterior-posterior approaches can achieve both decompression and stability reconstruction. A multicenter study indicates anterior approaches enable direct removal of compressive bone fragments with bone grafting fusion, while posterior long-segment fixation disperses stress and reduces implant failure rates.^[10]^ For AS patients with cervical fractures and SEH, combined anterior-posterior approaches can achieve both decompression and stability reconstruction. A recent meta-analysis by Peng et al including 215 patients from 11 studies demonstrated that while the posterior-only approach was associated with significantly reduced operative time and lower blood loss compared to combined approaches, there were no statistically significant differences in neurological improvement rates, complication rates, or mortality.^[11]^ This suggests that while combined surgery is more invasive, it does not necessarily yield superior neurological outcomes in all cases. However, another systematic review by Govindarajan et al provided additional nuance, indicating that while gross neurological improvement rates showed no significant difference between anterior and posterior approaches, the mean change in neurological function was significantly greater in patients undergoing posterior approaches compared to anterior approaches.^[12]^ Similarly, anterior approaches showed a lower mean change in neurological function relative to combined anterior–posterior approaches. These quantitative findings suggest that combined approaches may offer a biomechanical advantage that facilitates neurological recovery, particularly in cases with multi-column involvement and epidural hematoma compression. This case employed a combined anterior-posterior approach. Posterior surgery utilized ultrasonic bone scalpel for precise osteotomy, minimizing surrounding soft tissue damage while enhancing biomechanical stability through pedicle screw fixation, consistent with literature-recommended “360-degree fusion” strategy.^[13]^ The lead surgeon innovatively employed short-segment laminectomy decompression, utilizing a customized dural soft catheter combined with syringe irrigation to repeatedly flush and aspirate blood clots within the spinal canal. Substantial clots were evacuated, achieving effective decompression of the compressed dural sac while avoiding posterior bony structure destruction from extensive laminectomy. This balanced resolution of the conflict between adequate decompression and preservation of posterior spinal biomechanical stability provides valuable exploration for future minimally invasive laminotomy techniques involving windowed decompression with syringe-assisted irrigation through customized dural catheters to evacuate spinal hematomas. However, this procedure may increase cerebrospinal fluid leakage risks. Meticulous operative techniques with gentle manipulation were emphasized intraoperatively. The soft texture of the customized dural catheter effectively mitigated such risks. Future studies will expand sample sizes to rigorously investigate its safety and efficacy. AS patients have 2–3 times higher perioperative infection risk than normal individuals, potentially related to chronic immunosuppression and surgical trauma.^[14]^ However, the perioperative application of TNF-α inhibitors in AS patients undergoing major spinal surgery, especially in emergency settings complicated by SEH, remains a complex and contentious issue. The primary concern is the significantly increased risk of postoperative infections, as these agents cause systemic immunosuppression. AS patients already possess a baseline elevated risk of infection compared to the general population. Major spinal surgery involves substantial soft tissue dissection and implant placement, further elevating this risk.^[15]^ Current consensus generally recommends withholding biologic therapies prior to elective major surgery; the timing is based on the drug’s half-life, typically skipping 1–2 doses before surgery.^[16]^ Nevertheless, in acute traumatic scenarios like the present case, where the patient was not on regular biologic therapy and required immediate surgical intervention, the urgent need for neural decompression takes unequivocal precedence. The decision to initiate TNF-α inhibitors postoperatively must be carefully weighed against the individual’s infection risk, wound healing status, and the presence of any ongoing infection. In this case, given the extensive surgical trauma, elevated inflammatory markers (CRP: 11.99 mg/L), and the critical nature of preventing implant-related infection, TNF-α therapy was not initiated during the immediate perioperative period. The focus was instead on rigorous antibiotic prophylaxis and meticulous wound care. Early postoperative rehabilitation is equally crucial. Studies demonstrate that initiating passive joint mobilization within 24 hours postoperatively in AS patients reduces heterotopic ossification incidence.^[17]^ This patient exhibited gradual postoperative motor recovery but persistent saddle anesthesia, indicating the need for individualized rehabilitation plans to improve long-term prognosis. The timing of hematoma evacuation is closely related to neurological recovery. Animal studies show spinal cord compression exceeding 8 hours causes irreversible axonal damage.^[18]^ This patient underwent surgery within 24 hours post-injury, with lower extremity muscle strength recovering from grade 2 to grade 5 postoperatively, confirming the importance of early intervention. Additionally, chronic spinal cord ischemia in AS patients may affect recovery potential, while intraoperative neurophysiological monitoring helps assess surgical efficacy.^[19]^ Furthermore, successful management of such complex cases hinges on structured multidisciplinary collaboration. Our multidisciplinary team comprised spine surgeons, rheumatologists, radiologists, anesthesiologists, and rehabilitation specialists. The collaborative workflow began with radiologists providing urgent imaging interpretation to define fracture morphology and hematoma extent. Spine surgeons and anesthesiologists collaboratively devised an airway management and surgical plan, considering the potential for difficult intubation due to rigid cervical deformity. Rheumatologists advised on perioperative inflammatory control and the timing of biologic agent reintroduction. Postoperatively, rehabilitation specialists designed and implemented an individualized, phased exercise regimen starting within 24 hours, focusing on functional recovery while monitoring neurological status. This integrated multidisciplinary team approach ensured comprehensive care from diagnosis through long-term recovery.

## 4. Conclusion

In conclusion, for extensive posterior SEH in cervicothoracic regions, the technique of short-segment laminectomy decompression combined with the use of a customized dural catheter and syringe irrigation effectively evacuates distal spinal hematomas without compromising the integrity of posterior osteoligamentous structures. This approach, demonstrated in our case, achieved significant neurological recovery and was associated with favorable outcomes at the 2-year follow-up. Crucially, radiographic evaluation at 24 months postoperatively revealed well-maintained cervical alignment, no signs of hardware failure or migration, and importantly, no evidence of adjacent segment degeneration on static and dynamic radiographs. Specifically, there were no significant changes in cervical curvature (C2-C7 Cobb angle) compared to immediate postoperative measurements, and follow-up MRI showed no notable degenerative signal changes in the intervertebral discs at adjacent levels. Subsequent rehabilitation achieved substantial functional and sensory recovery, providing a valuable reference for managing similar cases. Furthermore, multidisciplinary collaboration remains essential for the management of AS patients with cervical fractures and SEH, emphasizing early imaging diagnosis, individualized surgical approaches, and comprehensive perioperative management. Future research should include larger cohorts and longer follow-up periods to further validate the long-term biomechanical stability and rates of adjacent segment disease associated with this minimally invasive decompression technique, and explore the potential role of biologics (e.g., TNF-α inhibitors) in reducing vascular fragility as well as the application of robotic-assisted surgery in complex AS spinal injuries.

## Author contributions

**Conceptualization:** Yongbo Li, Fuli Huang.

**Data curation:** Yongbo Li, Huilin Feng, Deyuan Chen.

**Methodology:** Yongbo Li.

**Project administration:** Deyuan Chen, Jianqiang Yan, Fuli Huang.

**Supervision:** Jianqiang Yan, Fuli Huang.

**Writing – original draft:** Yongbo Li.

**Writing – review & editing:** Huilin Feng, Deyuan Chen, Fuli Huang.
